# Facilitators of and Barriers to Integrating Digital Mental Health Into County Mental Health Services: Qualitative Interview Analyses

**DOI:** 10.2196/45718

**Published:** 2023-05-16

**Authors:** Xin Zhao, Nicole A Stadnick, Eduardo Ceballos-Corro, Jorge Castro Jr, Kera Mallard-Swanson, Kristina J Palomares, Elizabeth Eikey, Margaret Schneider, Kai Zheng, Dana B Mukamel, Stephen M Schueller, Dara H Sorkin

**Affiliations:** 1 Department of Medicine University of California, Irvine Irvine, CA United States; 2 Department of Psychiatry University of California, San Diego La Jolla, CA United States; 3 Altman Clinical and Translational Research Institute Dissemination and Implementation Science Center University of California, San Diego La Jolla, CA United States; 4 Child and Adolescent Services Research Center San Diego, CA United States; 5 Department of Psychological Science University of California, Irvine Irvine, CA United States; 6 Herbert Wertheim School of Public Health and Human Longevity Science University of California, San Diego La Jolla, CA United States; 7 The Design Lab University of California, San Diego La Jolla, CA United States; 8 Department of Public Health University of California, Irvine Irvine, CA United States; 9 Department of Informatics University of California, Irvine Irvine, CA United States

**Keywords:** digital mental health, mobile health, mHealth, implementation readiness, implementation science, qualitative analyses, mobile phone

## Abstract

**Background:**

Digital mental health interventions (DMHIs) represent a promising solution to address the growing unmet mental health needs and increase access to care. Integrating DMHIs into clinical and community settings is challenging and complex. Frameworks that explore a wide range of factors, such as the Exploration, Preparation, Implementation, Sustainment (EPIS) framework, can be useful for examining multilevel factors related to DMHI implementation efforts.

**Objective:**

This paper aimed to identify the barriers to, facilitators of, and best practice recommendations for implementing DMHIs across similar organizational settings, according to the EPIS domains of inner context, outer context, innovation factors, and bridging factors.

**Methods:**

This study stems from a large state-funded project in which 6 county behavioral health departments in California explored the use of DMHIs as part of county mental health services. Our team conducted interviews with clinical staff, peer support specialists, county leaders, project leaders, and clinic leaders using a semistructured interview guide. The development of the semistructured interview guide was informed by expert input regarding relevant inner context, outer context, innovation factors, and bridging factors in the exploration, preparation, and implementation phases of the EPIS framework. We followed a recursive 6-step process to conduct qualitative analyses using inductive and deductive components guided by the EPIS framework.

**Results:**

On the basis of 69 interviews, we identified 3 main themes that aligned with the EPIS framework: readiness of individuals, readiness of innovations, and readiness of organizations and systems. Individual-level readiness referred to the extent to which clients had the necessary technological tools (eg, smartphones) and knowledge (digital literacy) to support the DMHI. Innovation-level readiness pertained to the accessibility, usefulness, safety, and fit of the DMHI. Organization- and system-level readiness concerned the extent to which providers and leadership collectively held positive views about DMHIs as well as the extent to which infrastructure (eg, staffing and payment model) was appropriate.

**Conclusions:**

The successful implementation of DMHIs requires readiness at the individual, innovation, and organization and system levels. To improve individual-level readiness, we recommend equitable device distribution and digital literacy training. To improve innovation readiness, we recommend making DMHIs easier to use and introduce, clinically useful, and safe and adapting them to fit into the existing client needs and clinical workflow. To improve organization- and system-level readiness, we recommend supporting providers and local behavioral health departments with adequate technology and training and exploring potential system transformations (eg, integrated care model). Conceptualizing DMHIs as services allows the consideration of both the innovation characteristics of DMHIs (eg, efficacy, safety, and clinical usefulness) and the ecosystem around DMHIs, such as individual and organizational characteristics (inner context), purveyors and intermediaries (bridging factor), client characteristics (outer context), as well as the fit between the innovation and implementation settings (innovation factor).

## Introduction

### Background

Unmet mental health needs are consistently documented across age groups, diagnoses, and settings worldwide [1-3]. More than 80% of individuals with mental health challenges do not receive the treatment they need [4], which in the United States has resulted in an estimated 25 million Americans with mental health needs remaining untreated [5]. Higher levels of unmet needs exist among marginalized groups, including uninsured and low-income individuals [2]. This lack of treatment access and use is in part owing to system factors (eg, societal stigma, inequitable health policy, high out-of-pocket costs, long waitlists, and lack of access to mental health professionals) and individual factors (eg, self-stigma, perceived need, treatment beliefs, and psychological literacy) [6,7]. These factors are complex at the county, state, and federal levels, and their interacting pathways can all influence mental health equity [8], indicating a need to find scalable and cost-effective solutions that can change the status quo of systems of care.

Digital mental health interventions (DHMIs) represent a promising solution to address the growing unmet mental health needs by increasing access to care and reducing care disparities among marginalized groups [9-11]. DHMIs are web and mobile technologies that aim to assess, manage, and treat mental health conditions [12]. Considerable evidence for the efficacy and utility of DMHIs [13,14], especially for mood and anxiety disorders [15,16] exists. However, the sheer number of DMHIs outstrips scientific studies, and there is limited evidence on the evaluation of DMHIs in their implementation context [17,18].

Moving beyond efficacy, considerable work is underway to try to integrate DMHIs into the real-world settings. In this quest, an important consideration is that DMHIs are services, rather than products, and as such, their integration into organizational and community contexts requires mindful implementation efforts [19]. Various health and public mental health systems have attempted to use DMHIs as a cost-effective method for scaling access to care. For example, the Kaiser Permanente health system provided a suite of mobile apps to their members [20], and the city of Reno, Nevada, made Talkspace freely available to all of their >200,000 residents [21]. In the Kaiser Permanente example, considerable effort went into developing the clinical referral and self-care pathways and the training and support model for providers, demonstrating examples of active implementation support. However, in the city of Reno, after an initial 1-year rollout, with approximately 3100 residents taking advantage of Talkspace, the contract was not renewed [21], indicating that successful sustainment of such programs might be challenging. These efforts from health care systems and local behavioral health agencies suggest a need for developing a better understanding of the barriers and facilitators pertaining to DMHI implementation.

### Prior Work

Prior research has indicated that integrating DMHIs into clinical and community settings is particularly challenging because of barriers at different levels [22-24]. Individual perspectives, innovation characteristics, and related system-level contexts (eg, organization, neighborhood, county, and state levels) are critical factors contributing to implementation success [25]. Despite strong interest in DMHIs, the adoption rates among clients and providers have been low. In a study evaluating the effectiveness of mobile health provider training, less than half of the clinical providers reported integrating mobile health into their practice before training [26]. In another study of DMHIs examining referral pathways through a randomized controlled trial, fewer clients were reached through provider referrals than through direct-to-consumer communication (most commonly by email) [27], suggesting a need for understanding provider-level barriers to introducing DMHIs. Although the COVID-19 pandemic has accelerated the use of DMHIs [28,29], integrating DMHI into clinical practice continues to be challenging. At the system level, payment models for mental health care in the United States have resulted in additional barriers to the implementation of DMHIs [30]; time spent by providers, especially nonphysician health care providers (eg, nurse practitioners, psychologists, and social workers), on DMHI referrals is often not billable [31,32]. Prior research on this topic mostly focused on provider- and client-level barriers to and facilitators of implementing DMHIs [24,33-35], suggesting the need for understanding multilevel influences at the system, implementing organizational, and DMHI levels along with individual client and provider influences.

Thus, frameworks that explore multilevel determinants of implementation success can be useful for mitigating this gap in the field. Implementation science frameworks offer an opportunity to systematically assess such multilevel determinants. One such framework that is particularly suitable for examining DMHI implementation efforts is Exploration, Preparation, Implementation, Sustainment (EPIS) [36,37]. The EPIS framework, developed to guide systematic, multilevel, and context-sensitive assessments of implementation efforts across distinct phases, has been widely used [37]. Compared with other frameworks, the EPIS framework is relatively young yet widely used across implementation phases and domains, especially in mental health sectors and publicly funded service settings [37]. In addition, EPIS operationalizes key implementation influences across four major domains: (1) *inner context* factors (eg, organizational characteristics, leadership structures, and provider characteristics), (2) *outer context* factors (eg, fiscal mandates, policy drives, and incentive structures), (3) characteristics of the *innovation* being implemented (eg, the fit between a selected evidence-based practice and the training that providers have to receive to deliver the practice), and (4) *bridging factors* that impact or intersect the inner and outer contexts of implementation (eg, community-academic partnerships). A recent mixed methods study that examined the barriers to and facilitators of implementing DMHIs using the EPIS framework conducted by Lattie et al [22] revealed tensions surrounding technology, therapy, and organization and systems within a large community mental health service organization. Although the authors stated that this is representative of similar community organizations across the United States, further research is needed with larger sample sizes and more diverse publicly funded service contexts.

This paper used the EPIS framework to guide qualitative data collection and analysis with clinical staff, peers, and leadership across multiple counties in a collaborative project focused on DMHI exploration and implementation. The overarching aim of this study was to identify the barriers to, facilitators of, and best practice recommendations for implementing DMHIs across similar organizational settings according to the EPIS domains of inner context, outer context, innovation factors, and bridging factors.

## Methods

### The Help@Hand Project

This paper stems from a large state-funded project, Help@Hand, which explores the use of DMHIs as part of county mental health services. The Help@Hand project is a multiyear endeavor initiated in 2017 to improve the access to and impact of behavioral health care through DMHIs across 2 city and 13 county behavioral health departments in California, United States. DMHIs in Help@Hand fall under three categories: (1) peer chat or digital therapeutics, (2) virtual evidence-based therapy using an avatar, and (3) digital phenotyping using passive data collection. Although Help@Hand was launched with 2 technology vendors initially, various DMHIs have been added throughout the project to reflect the changing needs of counties and cities. A formative evaluation of this project is being conducted by a research team at the University of California, Irvine. Details of the evaluation are reported elsewhere [38].

**Figure 1:**
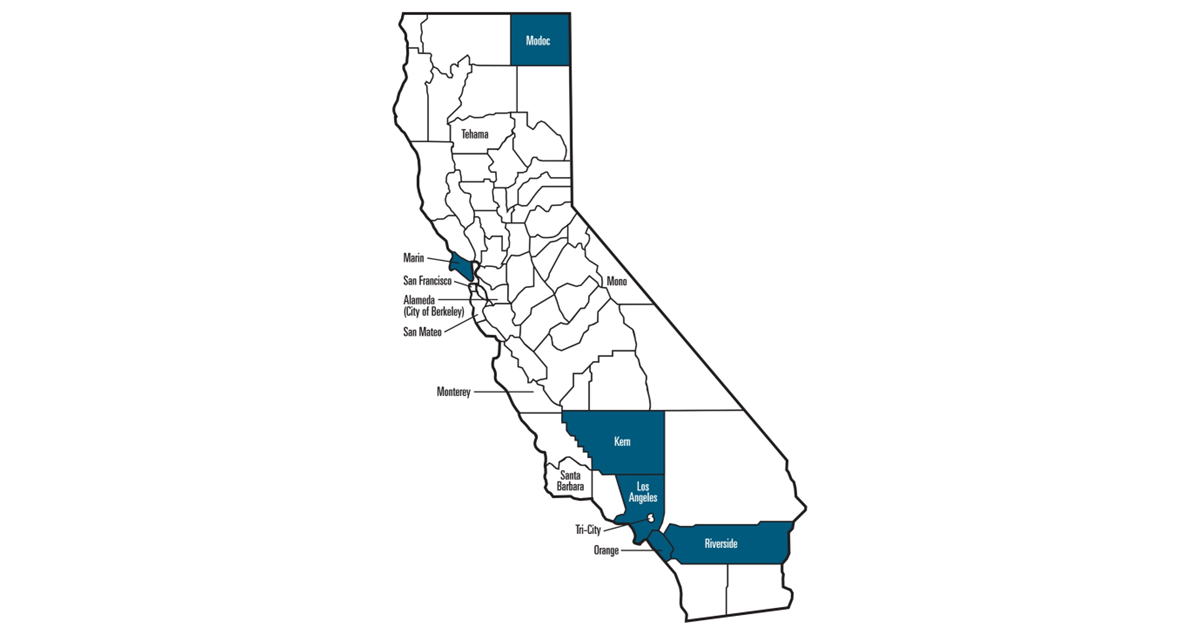
Geographical locations of the participating counties and cities. The counties or cities with annotations participated in the Help@Hand Project. Individuals from the counties that are highlighted contributed to the interview data in this study. Among the 6 included counties, Modoc County is considered rural according to the most recent delineation of the US Census Bureau.

The interview data presented in this paper were part of the quality improvement component of the evaluation. Interviews were conducted with clinical staff (ie, service providers, nurse interns, clinical supervisors, and community health workers), peers (ie, persons with lived experience with mental health challenges who were hired to support project planning and implementation), clinic leaders, project leaders, and county leaders from 6 California counties participating in the Help@Hand project (Table 1). Individuals who participated in the interviews were nominated by the leadership based on their knowledge of and experience in the project. Participation in the interviews was voluntary, and personal identifying information, including names, sociodemographic characteristics, and training backgrounds of the interviewees, was not collected. Participating counties were spread across different parts of California (refer to Figure 1 for a map), consisting of both urban and rural settings, with population sizes ranging from 8700 to 9.9 million. According to the most recent delineation of the US Census Bureau, Modoc County is considered rural, and the other 5 counties are considered urban [39]. A total of 69 interviews were included in our analyses.

**Table 1:** Participant information.

County	Early implementation, n (%)		After implementation, n (%)	
	Interviews (n=33)	Participant role (n=33)	Interviews (n=36)	Participant role (n=38)
Los Angeles	6 (18)	4 (12) service providers 2 (6) clinic leaders	9^a^ (25)	7 (18) service providers 2 (5) clinic leaders 1 (3) clinical supervisor
Kern	15 (45)	15 (45) service providers	2 (6)	2 (5) project leaders
Modoc	9 (27)	9 (27) service providers	2^a^ (6)	2 (5) project leaders 1 (3) clinic leader
Orange County	3 (9)	2 (6) clinical supervisors 1 (3) service provider	N/A^b^	N/A
Riverside	N/A	N/A	11 (31)	11 (29) peers
Marin	N/A	N/A	12 (33)	4 (11) community health workers 1 (3) peer 5 (13) nurse interns 1 (3) county leader 1 (3) clinic leader

^a^Included 1 interview jointly conducted with 2 interviewees.

^b^N/A: not applicable.

### Ethical Considerations, Informed Consent, and Compensation

Our institutional review board deemed that this work was exempt from human participant research approval (University of California, Irvine Institutional Review Board# 2019-5406). We obtained verbal consent from all the participants before the interviews. The study data were deidentified before analysis. No direct compensation was provided for the interviews. However, for in-person site visits, we provided lunch for the entire clinical staff, regardless of whether they completed an interview, in appreciation of their participation in the evaluation.

### Interview Procedures

The development of the semistructured interview guide was informed by expert input regarding relevant inner context, outer context, innovation factors, and bridging factors in the exploration, preparation, and implementation phases of the EPIS framework. We also piloted the interview guide with the project leadership in each county to ensure that it would be understandable and relevant to their implementations. Between June 2018 and March 2021, we conducted 33 early implementation (ie, approximately 1 month after the products were initially introduced) interviews and 36 postimplementation (ie, after the full-scale deployment of the products in the county and at least 6 months after the early implementation interviews) interviews. Independent interviewers with expertise in digital mental health and implementation science (SMS, NAS, and KM-S) conducted in-person (before COVID-19 pandemic) or web-based (owing to the need for social distancing) interviews, most of which included 2 interviewers (ie, 1 notetaker and 1 interviewer) and 1 interviewee. The assignment of interviewers to an interviewee was determined based on the availability of interviewers. Each interview took approximately 30 minutes to complete. A general interview guide template is provided in Multimedia Appendix 1.

### Coding and Analytical Procedures

To analyze the qualitative data collected, we followed a recursive 6-step process outlined by Braun and Clarke [40]. A combination of deductive and inductive approaches was used for thematic analysis. For phases 1 and 2 (becoming familiar with the data and generating initial codes), the interviews were audio recorded, transcribed, reviewed, and anonymized for transcription accuracy before coding. The participants were asked not to include identifiable information in their responses. The initial development of the codebook was guided by the EPIS framework using the constructs assessed in the interviews. All coders (KM-S, EC-C, JC, and KP) were provided with an overview of the EPIS framework and then trained in qualitative coding best practices and the use of the coding software (NVivo [QSR International]). Coders collaboratively coded 4 interviews using a spreadsheet to familiarize themselves with the data and codebook. They also discussed revisions to the codebook (eg, clarification of domain definitions). After the codebook was finalized, the coders were assigned interview transcripts to conduct independent coding. They met weekly during this process to resolve any discrepancies. Discrepancies that were not resolved between 2 coders were discussed with 2 domain experts (SMS and NAS), and the final codes were assigned upon consensus. After the initial 10% of all the transcripts were double coded, 3 of the trained coders (EC-C, JC, and KP) completed the coding of the remaining transcripts using NVivo. For phases 3 to 5 (searching for, reviewing, defining, and naming themes), R (R Foundation for Statistical Computing) was used to calculate the frequency of the codes by EPIS domains to obtain an overview of the code distribution and to identify which codes were more common. We constructed salient themes defined as “captures something important about the data in relation to the research question, and represents some level of patterned response or meaning within the data set” [40]. Our process of reviewing, defining, and naming themes was supported through collaborative discussion with the larger research team regarding codes believed to be impactful barriers to, facilitators of, and recommendations for DHMI implementation. For phase 6 (producing the report), XZ, SMS, and NAS engaged in documenting the themes described in this paper.

## Results

### Overview

Individual characteristics (inner context), organizational characteristics (inner context), innovation characteristics and fit (innovation factor), and client characteristics (outer context) were among the commonly mentioned facilitators and barriers. Each of these domains comprised of >5% of the total codes and was mentioned by interviewees from all 6 counties. More than 50% of the recommendations found in the data (in terms of the percentage of codable chunks) focused on innovation characteristics. Recommendations regarding organizational characteristics were also commonly mentioned. The frequencies of EPIS codes are presented in Multimedia Appendix 2.

We identified 3 main themes that aligned with the EPIS framework related to the overall implementation experience of the participating Help@Hand counties. These 3 themes all centered on readiness: the readiness of individuals (in this case, clients), the readiness of the innovations (in this case, the DMHIs implemented), and the readiness of the organizations and systems (in this case, the clinics or counties where DMHIs were being deployed). Individual-level readiness included the extent to which the clients had the tools (eg, smartphones) and skills (digital literacy) necessary for using DMHIs. Innovation-level readiness referred to accessibility, usefulness, safety, and innovation fit. Organization- and system-level readiness pertained to the extent to which providers and county leaders collectively held positive views about DMHIs, as well as the extent to which the infrastructures of organizations (eg, staffing) and systems (eg, payment model) were appropriate for DMHIs. We present the themes, EPIS codes, and example quotes in Table 2.

**Table 2:** Qualitative themes from Exploration, Preparation, Implementation, Sustainment (EPIS) coding.

Themes, subthemes, and EPIS domains				Example quotations
**Individual readiness**				
	**Technology access**			
		Outer context: client characteristics	“The biggest problem with our clients in community mental health was that they didn’t have access to the type of phone that could use the app.”	
		Outer context: service environment	“Give them a smartphone. Give them free telephone service for a month or so with the app on it and let them use it. That’s just an economic incentive for using the program.”	
		Inner context: organization characteristics	“We want to have kiosks. We want to have [kiosks] available everywhere.”	
	**Digital literacy**			
		Outer context: client characteristics	“folks who are comfortable, obviously, with technology would respond very, very positively, and you’d have a [large] population who would feel very comfortable accessing care through a tablet or a phone.”	
		Outer context: client advocacy	“I actually developed a telehealth equity toolkit with trainings and links to different things for providers to use in trying to help older adults and individuals with disabilities bridge that digital divide.”	
		Bridging factors: purveyors and intermediaries	“If we wanted to hit folks without the digital literacy, it’d have to be so hands-on and so much more comprehensive than we’re staffed.”	
**Innovation readiness**				
	**Ease of use and introduction**			
		Innovation factors: innovation characteristics	“I think a lot of people got irritated with the notifications. I just turned my notifications off, but I think [too many notifications] turned people off.”	
	**Clinical usefulness**			
		Innovation factors: innovation characteristics	“Feeling anxious, here are some tools you can use. Need somebody to listen, you’re having some challenging thoughts, you have somebody to talk to. I think that easy accessibility and support [timely mental health support] is great...to highlight.”	
	**Safety**			
		Innovation factors: innovation characteristics	“We actually tested [a DMHI^a^] on some of the things like giving it word prompts, then see what it would do, and it wasn’t performing the way it was supposed to. It didn’t recognize the word suicide. It didn’t recognize the word homicide. There were other scenarios that involved active shooters that it didn’t recognize?”	
	**Innovation fit**			
		Innovation factors: innovation fit	“They [clinical staff] were freaking out because they were getting messages. They had to respond back, take the time out of their day, make a response, enter a note, fax it back, and it was stressing some people out.”	
		Inner context: individual characteristics	“I work with a lot of children, some of whom have Asperger’s, and apps is not what we need.”	
		Innovation factors: innovation characteristics	“It would be good to have client feedback in an area of—even on the phone or in the app where a client could provide feedback if they wanted to about how the app is going.”	
**System readiness**				
	**Compatibility**			
		Inner context: organization characteristics	“But my sense is that some of them are really into it, and some of them really aren’t. A lot of people are indifferent, unfortunately.”	
	**Privacy and trust**			
		Outer context: client characteristics	“And the other thing, too, is trust. These clients have experienced a lot of abuse from authority, police, judges, and other Big Brothers in society. And the idea of having an app that monitors your activity seems like another Big Brother.”	
	**Infrastructure**			
		Inner context: organization characteristics	“Our IT Department recommended iPads. We didn’t have the budget for iPads. There was a lot of logistical challenges using the Samsungs.”	
		Bridging factors: purveyors and intermediaries	“I think that would be something is making sure that we [providers] get enough training and consistent training, if we get updated and how to use it effectively and appropriately.”	
		Outer context: leadership	“Even though the Department of Health Services approved the use of HipaaBridge, it took four years before they [leadership] would approve it”	
		Outer context: interorganizational environment and networks	“communicating well between vendors and a private sector and translating that into county speak.”	

^a^DMHI: digital mental health intervention.

### Theme 1: Individual Readiness

#### Technology Access

Access to technology was mentioned as a factor that influenced implementation success. Some service providers reported that smartphones were widely used by their younger clients, whereas other service providers mentioned that their current clients in community mental health settings usually did not own smartphones (outer context, client characteristics). One service provider stated, “The biggest problem with our clients in community mental health was that they didn’t have access to the type of phone that could use the app.” Service providers also mentioned limited hardware capability (eg, low memory) on clients’ phones and reliance on Wi-Fi (and the lack thereof) as smartphone-related barriers to the uptake and use of DMHIs. Some of the counties participating in Help@Hand had already undertaken efforts to increase access to technologies (inner context, organizational characteristics). Such efforts can help facilitate the success of deploying DMHI products, as they provide useful infrastructure for people to use DMHIs. One of the service providers described, “And so when we started out, we developed iPad kiosks in our lobby as free places for people to go in and enroll in the system.” In counties where this had not yet happened, they recognized the value that increasing technology access could have for this project, as conveyed by a peer support specialist from another county: “We want to have kiosks. We want to have [kiosks] available everywhere.” Similarly, another service provider suggested that providing technologies (outer context, service environment) could both address barriers and incentivize DMHI adoption: “Give them a smartphone. Give them free telephone service for a month or so with the app on it and let them use it. That’s just an economic incentive for using the program.” In addition to providing technology, a peer mentioned a need for offline features on DMHIs (eg, “accessible without needing to be connected to Wi-Fi or accessible without using too terribly much data or creating roaming charges”).

#### Digital Literacy

Digital literacy in general and the levels of comfort with DMHIs in particular were identified as key factors influencing DMHI adoption [41]. In our context, digital literacy is broadly defined as the ability to find, evaluate, communicate, and use mental health information through websites and mobile apps [42]. Service providers, community health workers, and peers described different levels of digital literacy among clients (outer context, client characteristics). Some clients had basic levels of digital literacy, as described by one of the service providers: “folks who are comfortable, obviously, with technology would respond very, very positively, and you’d have a [large] population who would feel very comfortable accessing care through a tablet or a phone,” whereas others lack the basic digital skills to use DMHIs, as noted by a peer: “but just the process of creating a [user]name and password and knowing where to touch on a screen is horrendous for someone who’s never done it.” The lack of digital literacy and confusion about DMHI data reduced providers’ motivation to register as a user of DMHIs themselves and introduce the tools to clients. Service providers, peers, and community health workers mentioned the issue of the lack of digital literacy, which was more common among clients who were older, from lower-income backgrounds, experiencing homelessness, unable to read or write, or monolingual non-English speakers. For example, one of the community health workers mentioned, “a lot of older people like myself, first navigating the web and getting to these different apps, downloading stuff is not easy.” In light of a lack of support and training, service providers, clinic leaders, and project leaders were trying to improvise by creating content that could prepare their clients to use DMHIs (outer context and client advocacy efforts). For example, one of the project leaders described what they did to facilitate the implementation: “I actually developed a telehealth equity toolkit with trainings and links to different things for providers to use in trying to help older adults and individuals with disabilities bridge that digital divide.”

Both clinical staff and county leadership recognized a need for broad education for digital literacy and support for specific DMHI features. One of the county leaders mentioned, “[We] are getting some peer requests, especially among older adults, for education around computer literacy and digital literacy” (outer context, client characteristics). Our participants also suggested more staffing and workforce support to ensure *accessible* and *digestible* digital literacy training (bridging factors, purveyors and intermediaries). For instance, one of the county leaders mentioned, “if we wanted to hit folks without the digital literacy, it’d have to be so hands-on and so much more comprehensive than we’re staffed.” Our data suggested that one potential way of improving clients’ digital literacy was to leverage provider support (inner setting, individual characteristics), such as carving out in-session time to support the initiation and sustained use of DMHIs, as mentioned by one of the clinical supervisors: “If we could take an extra step of actually downloading [DMHIs] together or something, that might actually help. If I said in session download this right now and then I said I’m going to check on you next week to see how it works.” In addition to human support, built-in support, such as tutorials available through apps, was also recommended. One of the nurse interns suggested “an option to have a navigation tutorial, just something to ask [clients] if they need help navigating through things.”

### Theme 2: Innovation Readiness

#### Ease of Use and Introduction

The perceived ease of use and introduction of a DMHI was identified as an innovation characteristic (innovation factor) influencing implementation. Service providers and peers discussed challenges related to navigating websites, setting up accounts, using app-based keyboards that are often lagging or have inaccurate autocorrection features, and different experiences in using the interventions across DMHI versions and devices (eg, Android vs iPhone [Apple Inc]). These barriers led to enrollment difficulties and contributed to providers’ struggles with introducing and explaining a DMHI tool to clients. One of the service providers stated, “So [information provided by DMHI] needs to be readable, and their website’s not as readable, understandable, and easy to access as it should be. And that’s just one of the major problems.” In addition, certain innovation characteristics can temper clients’ and service providers’ enthusiasm for continued use, such as too many notifications and a lack of timely feedback. One of the service providers reflected, “I think a lot of people got irritated with the notifications. I just turned my notifications off, but I think [too many notifications] turned people off.” Multiple clinical staff members and county leaders desired better DMHI usability (eg, “simple,” “website easy to read,” and “easy to understand”), which could increase the adoption of DMHIs for individuals with low digital literacy. For instance, one of the service providers recommended the following: “making it more simplified so that it’s not so technological or advanced, especially for those that aren’t familiar with phones.” Nurse interns also mentioned specific features that could increase providers’ ease of introducing the tool (eg, “having automated instructions” and “having sample scripts”).

#### Clinical Usefulness

Perceived clinical usefulness was another innovation characteristic (innovation factor) influencing clients’ willingness to start and continue using DMHIs. Availability, convenience, and anonymity were identified as facilitators. Providers mentioned that DMHIs helped supplement their limited time with clients in session and remind clients about treatment goals. Easy-to-access data visualization of goals and symptoms, self-management care tools (eg, mindfulness techniques), and 24/7 support were identified as particularly helpful when a provider was not available. For instance, one of the service providers stated, “Feeling anxious, here are some tools you can use. Need somebody to listen, you’re having some challenging thoughts, you have somebody to talk to. I think that easy accessibility and support [timely mental health support] is great...to highlight.” Furthermore, nonverbal communication modalities and the anonymous nature of certain DMHIs can facilitate help seeking among clients who experience abuse, societal stigmatization, and self-stigmatization. One of the peer support specialists described this as follows: “The convenience definitely. A lot of [clients] out there who are in need of support could be in dangerous situations where, like, a verbal phone call could really put their well-being at risk, right, when it comes to, like, abusive relationships or things that. So, definitely the convenience and the anonymity of the program.”

Meanwhile, despite the mentioned potential of DMHIs to supplement traditional treatment, multiple participants mentioned that DMHIs could not replace face-to-face services when building trust and connections. A service provider commented the following during an early implementation interview: “You don’t have a face-to-face communication. I mean, that’s the most important. I feel like a person may be able to express themselves or have more connection with someone in person versus on an app.” Nonverbal cues (eg, eye contact) and tones of speech, which are often not captured in chat rooms and texting, are important to understand contextual factors, as expressed by one of the peer support specialists during their postimplementation interview: “I was also anxious about being able to connect with people because, as a peer operator [peer], that the core of what we do and who we are is connection. Mutuality you can still do, but I thought, well, without the eye-to-eye contact or hearing their expressions and the tone of voice, will we really be able to connect? And that was my major concern.”

To reduce hesitation and increase confidence, multiple clinical staff members expressed the need to know whether there was clinical evidence demonstrating the effectiveness and usefulness of the specific DMHIs selected before introducing them to their clients. For example, one of the service providers mentioned the following in their early implementation interview: “There’s constantly research coming out about what’s going to be most helpful, so kind of updating with that information or with recent studies.” Clinical supervisors and service providers also mentioned a few specific features that could potentially increase the clinical utility of DMHIs, including supporting progress monitoring (eg, “what we need is...give us a glance into how people are doing over the course of time, as opposed to what they say at the moment that they’re in the clinic”), providing real-time feedback (eg, “it has to be real time if it’s going to be useful for me as a clinician”), and linking clients with resources timely (eg, “there needs to be...links for resources”).

#### Safety

Safety was another innovation characteristic (innovation factor) identified; concerns about the safety of DMHIs were barriers to implementation. Clinical staff reported situations in which digital communication modalities (eg, artificial intelligence–assisted chat and peer support) may not be safe enough for crisis management. For example, one of the service providers explained, “we actually tested [a DMHI] on some of the things like giving it word prompts, then see what it would do, and it wasn’t performing the way it was supposed to. It didn’t recognize the word suicide. It didn’t recognize the word homicide. There were other scenarios that involved active shooters that it didn’t recognize?” Furthermore, service providers also shared concerns about inappropriate web-based interactions that could take place on peer support platforms and chat rooms; one of the service providers stated, “But these chat rooms are not monitored, and so anyone can pop on and say a number of horrible things, and no one’s there to monitor that behavior. And we didn’t know that.” To enhance safety features, providers recommended monitoring chat forums for bullying and abuse and creating escalation plans and proper referral pathways to suicidal hotlines when applicable. For instance, one of the service providers stated, “[DMHI] needs to build in better safeguards in terms of if conversations go haywire. They need to create an escalation plan.”

#### Innovation Fit With Current Client Populations

The perceived fit of the innovation with the current client populations is related to innovation factors and inner context. Providers and leadership included in our analyses shared that innovation fit varied according to the providers’ practice settings (inner context, individual characteristics). Providers’ perceptions of the innovation’s fit with clients’ global functioning or age (eg, the older population, individuals with severe mental illness, and youth with autism spectrum disorders) were identified as a barrier. For example, one of the service providers commented, “I work with a lot of children, some of whom have Asperger’s, and apps is not what we need.” Difficulty with interpreting data, which is associated with client characteristics (eg, educational background and digital literacy), was another barrier to innovation fit. For example, data visualization of treatment progress (eg, symptom change) is useful only when clients are interested in and able to understand the data. Multiple providers recommended features related to personalization and catering to client preferences (innovation factor, innovation characteristics). For instance, one of the service providers mentioned, “We would want apps that have the flexibility and the ability to turn on or off different [features].” In addition, multiple clinical staff members recommended gathering client feedback to understand and meet client needs, as exemplified by another service provider’s statement: “it would be good to have client feedback in an area of—even on the phone or in the app where a client could provide feedback if they wanted to about how the app is going.” The importance of meeting the needs of historically marginalized groups was emphasized in our data. For instance, multiple providers recommended adding or improving the Spanish features of the DMHIs to meet the needs of Spanish-speaking clients. One of the nurse interns stated, “it was better to hear [a video feature] in Spanish rather than having to follow the captions and watch the video at the same time.”

#### Innovation Fit With Current Workflows

The perceived fit of the innovation with the current workflows is another innovation factor influencing implementation. The perceived relative advantage of DMHIs (eg, compared with traditional paper worksheets) led to different opinions about their fit with the current clinical workflow. Some service providers found DMHIs as a helpful and convenient alternative to paper-pencil worksheets (eg, “I like how much more accessible [the digital format] is for the clients. You know, everyone loses paper. It could be a little bit more private as opposed to a piece of paper.”). Other service providers found it difficult to incorporate DMHIs into their existing clinical practice; for example, using digital tools within sessions can be distracting for clients (eg, “When the technology piece is involved—and I’ve noticed this in our groups as well when we’re checking the diary cards—that having this out feels like there is a disconnect in terms of the clinical process that’s happening in the room”). The perceived burden of using a tool was mentioned as a common reason for poor fit with the current clinical workflow. One of the service providers recalled, “they [clinical staff] were freaking out because they were getting messages. They had to respond back, take the time out of their day, make a response, enter a note, fax it back, and it was stressing some people out.”

Clinical staff and leadership recommended features related to the improvement of the efficiency of their existing clinical tasks. For example, one of the service providers mentioned, “I would love for there to be an improvement in kind of the logistics and general running of the client workflow. So if there’s a way to have clients be able to complete outcome measures.” Service providers and nurse interns recommended features that could facilitate provider-client communication. One of the nurse interns mentioned, “I was hoping it would be a little bit nursing-related, more assessments, or even just therapeutic communications with these people just because of the pandemic.”

### Theme 3: System Readiness

#### Compatibility

Clinic leaders, peers, and service providers all mentioned that the compatibility between the shared values and the innovation within the organization and community is a determinant (inner context, organizational characteristics). A shared vision and enthusiasm for DMHIs were identified as facilitators of the introduction and use of DMHIs. In our analyses, sentiments toward DMHIs differed by organization and context, ranging from a clear lack of shared vision (eg, “But my sense is that some of them are really into [DMHI], and some of them really aren’t. A lot of people are indifferent, unfortunately”) to highly consistent vision and buy-in (eg, “We were all excited about [DMHI], and everybody, you know, in the meetings, we would talk about it, and so much positive”). Ongoing collaborative relationships and open communication with technology vendors, as well as clear expectations and supportive leadership within an organization, contributed to shared enthusiasm about DHMI implementation and improved implementation readiness. The perception that DMHI implementation was a low priority or lower in priority compared with other organizational initiatives coupled with limited funding resources contributed to low rates of provider buy-in (eg, “the general attitude I’ve heard is either is [implementation of DMHI] actually that helpful with information and couldn’t this money be put more effectively in other areas”).

#### Privacy and Trust

Privacy concerns, which were shared by multiple clinical staff members, emerged as barriers to provider referrals and client engagement. Service providers shared concerns about data misuse and privacy violations, which could be detrimental to their provider-client relationship. Service providers and community health workers also highlighted that data collection by a mobile app could be perceived as a threat to clients who had been involved in the legal system and clients who experienced substance abuse problems (outer context, client characteristics). For example, one of the service providers commented the following during their early implementation interview: “the other thing, too, is trust. These clients have experienced a lot of abuse from authority, police, judges, and other Big Brothers in society. And the idea of having an app that monitors your activity seems like another Big Brother.” One of the recommendations mentioned by service providers was to increase transparency regarding data use: “explicitly state how the information is going to be used and not used. It is also when they get on that site, when the clients are on that site, one of the first things they should become aware of is how this information is going to be used, and that’s not something to negotiate.” Other service providers recommended ensuring that data servers were secure and that DMHIs were Health Insurance Portability and Accountability Act (HIPAA)–compliant when applicable.

#### Infrastructure

Infrastructural problems at the organization (eg, inadequate technology, resources, training, and staffing) impeded implementation. During postimplementation interviews, multiple clinical staff members reported not receiving appropriate technology (eg, software and devices) from their organizations (inner context, organizational characteristics); examples of such concerns are a delay in getting a test account, not receiving devices that their IT department recommended, not having a work phone, and not having a designated office space with a computer. Providers mentioned that the lack of funding was a contributing factor to the technology access barrier. For example, one of the project leaders mentioned, “our IT Department recommended iPads. We didn’t have the budget for iPads. There [were] a lot of logistical challenges using the Samsungs.” The lack of appropriate DMHI-specific training contributed to confusion about specific DMHI features and related regulations (eg, HIPAA). For instance, one of the service providers commented the following: “there hasn’t been a lot of institutional support in order to do so just because of privacy violations—we were trying to respect HIPAA—even just being able to access programs, apps for free in order to support our clinical work in a way that’s efficacious and effective.”

Related to infrastructure improvement at the organizational level, multiple service providers recommended involving peers (bridging factors and purveyors, intermediaries) and providing ongoing organizational support related to technology in general (eg, IT support) and specific to the DMHI selected (eg, feature-specific training and step-by-step instructions; inner context, organizational characteristics). For example, one of the service providers mentioned, “I think that would be something is making sure that we [providers] get enough training and consistent training, if we get updated and how to use it effectively and appropriately.” Such training was mentioned as essential to building knowledge and confidence among service providers so that they were “more comfortable in explaining” DMHIs to their clients. In addition to providing provider-oriented training, multiple providers recommended involving peers. For instance, one of the project leaders stated, “there were a few counties that always had peers working...And I think [peer involvement] is, of all the things...the best thing.”

Infrastructural barriers were also mentioned at the system level, including staffing and payment models (inner context, organization and individual characteristics), as well as the process of leadership approval (outer context, leadership). Competing time demands were identified as a barrier to the uptake of and engagement with DMHIs across counties in our analyses, suggesting a lack of readiness in the staffing structure. Supervisors described the competing time demands of clinical staff, including administrative tasks, caseloads, and training. Peers, nurse interns, community health workers, and service providers reported having limited bandwidth to learn about and use DMHIs because of high caseloads and competing priorities. For example, one of the nurse interns stated, “If I’m going to be completely honest, it was only between two choices, COVID contact tracing or the Help@Hand project.” The current reimbursement model was identified as another barrier at the system level; most DMHIs are not billable, as described by one of the service providers described: “I think that one hiccup that we’re having is the perception that we cannot utilize [DMHI] as a tool to provide a billable service, so we can’t integrate [DMHI] into care at that level that makes it kind of compliant and part of the treatment plan.” Additional infrastructural issues mentioned in our data included a lengthy process of leadership approval (eg, “Even though the Department of Health Services approved the use of HipaaBridge, it took four years before they [leadership] would approve it”).

Multiple service providers mentioned the need for a more integrated care model and an enhanced integration of DMHIs into the existing clinical information systems. For example, one of the service providers mentioned, “If we can have an infrastructure change, the integration of that mind health [mental health] into our electronic health record [EHR] to create a more holistic view of our clients in more real time.” Integrating DMHIs into the electronic health record may facilitate timely communication between service providers and clients, as mentioned by another service provider: “It would be mind-bogglingly awesome if there was a way to get a notification in the EHR system, electronic health record system, saying, So-and-so has made this comment in the application, and we get a push notification or some kind of message that alerts us.” In addition to improving communication in clinical settings, multiple service providers and county leaders suggested better communication between integration sites and DMHI vendors (eg, “communicating well between vendors and a private sector and translating that into county speak”; outer context, interorganizational environment and networks). Another service provider made a staffing suggestion to enhance the communication between the health sector and technology vendor: “having a designated person from [technology vendor] that we can connect with that can answer our questions when we have them, and that person to streamline, I guess, information or questions from our end [county behavioral health] to [technology vendor].”

## Discussion

### Principal Findings

#### Overview

This paper presents the facilitators of, barriers to, and recommendations for the implementation of DMHIs through the lens of the EPIS framework. Empirical data were collected via interviews with clinical staff and county leaders across multiple counties in California in a state-wide DMHI project. The results showed that successful implementation requires readiness at the individual, innovation, and organization and system levels. Examining these facilitators and barriers and synthesizing recommendations allowed us to identify the following key lessons for implementing DMHIs.

#### Individual Readiness

Although 85% of US adults own a smartphone [43], the lack of technology access observed among clients in community mental health settings is an important consideration for the provision of equitable care and successful implementation of DMHIs. Clients’ smartphone ownership varies by their age, global functioning, and geographical location. Recent data suggested that clients with socioeconomic disadvantages, those with severe mental health illness, and those of older age are less likely to own a smartphone [43,44]. Increasing technology access requires understanding client characteristics and improving service requirements in the outer context. In our analyses, service providers and peers mentioned the importance of equitable device access and initiated efforts to improve technology access, such as establishing iPad kiosks. One of the service providers we interviewed also recommended incentivizing individuals to use DMHIs by providing them with smartphones preloaded with DMHIs. This approach was used by Schueller et al [45] in a feasibility trial for young adults experiencing homelessness; however, the associated costs warrant additional considerations for funding before the large-scale implementation of this strategy in the public sector.

In addition to access to technology (eg, smartphones), clients need skills and knowledge to make an informed choice of using DMHIs. Thus, it is paramount to understand individuals’ current level of digital literacy in the outer context and leverage purveyors and intermediaries to provide appropriate digital literacy training. Although our study included diverse perspectives from various stakeholders, including clinical staff, peers, clinic leaders, county leaders, and project leaders, we do not have information about the adoption of DMHIs from clients’ perspectives. Nonetheless, multiple service providers mentioned that older age, lower income, homeless status, and limited ability to read or write in English might have contributed to the low levels of digital literacy and hence difficulties in using DMHIs among their current clients. Ignoring such sociodemographic differences in digital literacy may increase the disparities in mental health care [10,11]. To address this problem, our data highlighted the importance of leveraging provider support to provide *accessible* and *digestible* digital literacy training (both broad and DMHI feature specific). This finding is similar to the results from a systematic review conducted by Vis et al [24] suggesting that “capacities and means” required for clients to use and providers to recommend DMHIs were documented as the determinants of the acceptance of DMHIs. Beyond engaging and empowering clinical staff, involving peers who have similar lived life experiences in supporting digital literacy training is also recommended to enhance the accessibility and digestibility of digital literacy training and other DMHI support [46].

#### Innovation Readiness

In addition to their availability, convenience, and anonymity, DMHIs need to have a range of other innovation characteristics, such as ease of use, clinical usefulness, and safety. As recommended by the clinical staff in our data, having automated instructions, sample scripts, and built-in tutorials may help make a DMHI easier to introduce and use. Developing materials for supporting providers’ decision-making regarding referrals of DMHIs and clients’ decision-making regarding the use of DMHIs may be particularly helpful. For example, Kaiser Permanente designed tear sheets that providers use to recommend mobile apps to their clients; the 1-page information sheet is available in paper and digital formats and includes information about content, information about functionalities, and “when to use” information pertaining to different DMHIs [20].

Perceived clinical utility differs by clinical context. It is well recognized in the literature that DMHIs have the potential to supplement the limited time clients have with mental health professionals because of their availability, convenience, and anonymity [12,19,22]. The providers in our study shared that DMHIs were particularly helpful when a professional was not available (eg, outside regular business hours) and highlighted important DMHI features, including progress monitoring and timely communication between sessions. That said, the value of face-to-face interactions was emphasized, and the preference for face-to-face care was a common barrier [22,23]. Although some studies suggested that telehealth yielded comparable clinical outcomes [47], clinical efficacy does not necessarily translate into acceptability and effectiveness. Differences in perceived relative advantages may result from various levels of clients’ and providers’ openness to DMHIs and different clinical contexts (eg, individual vs group therapy and treatment modalities), highlighting the importance of considering context-specific factors during DMHI implementation [25]. In addition, it is important to avoid homogenous assumptions about the clinical utility of DMHIs [18]. Therefore, we recommend *examining* the effectiveness and usefulness of specific DMHIs and their features and *communicating* these to clinical staff*.* In a similar vein*,* Graham et al [27] recommended reviewing DMHI evidence and content during the exploration stage, selecting DMHIs with demonstrated effectiveness, and creating and distributing educational materials about the DMHIs during the preparation and implementation phases.

Improving innovation fit to align a DMHI with the needs and priorities of the current client populations and provider workflows requires an understanding of client characteristics (outer context), innovation characteristics (innovation factor), and the fit between the 2 (innovation factor). It is paramount to understand the unique facilitators of and barriers to the use of DMHIs among marginalized clients in the outer context, such as individuals involved in the legal system and monolinguistic communities, to meet their needs [10]. The providers we interviewed mentioned that some clients found the digital format helpful when organizing treatment-related materials, whereas others (eg, clients of older age) might prefer paper-and-pencil formats. Having features (eg, diary cards) that are available in both digital and paper formats may help meet the needs of diverse client groups. In addition, ongoing tailoring and adaptation of the existing tools are necessary. For example, the providers who participated in our interviews identified the need to provide training in Spanish and translate app-related materials into Spanish. Furthermore, the innovations themselves need to be designed in ways that incorporate feedback from core populations as well as the implementation strategies used to reach such populations. To improve the innovation’s fit with provider workflows, DMHIs must prove to be a viable solution to solve problems or improve the efficiency of the existing workflows rather than add new tasks or disrupt workflows [22]. The providers in our study recommended specific DMHI features that could address the problem of a lack of communication and feedback between providers and clients.

#### System Readiness

Excitement about DMHIs is common [20,22,27], but the level of enthusiasm may vary to a great extent across practice settings, thus requiring a better understanding of the organizational characteristics in the inner context. Understanding and potentially cultivating enthusiasm in organizations may help ensure that organizations are ready to implement a specific DMHI tool. One consideration is the compatibility between a specific DMHI (or DMHIs generally) and the organizational culture (eg, general openness to try new technology). In our study, sentiments toward DMHIs were mixed, ranging from skepticism and a lack of buy-in regarding DMHIs from some providers to highly consistent enthusiasm and buy-in from all providers. These differences might reflect organizational and county-level differences related to factors such as budget, resources, rurality, clients’ diagnostic profiles, and practice settings. It may be particularly helpful for organizations and county behavioral health departments to conduct needs assessments among the various invested parties (eg, county leaders, clinic leaders, project leaders, peers, community health workers, nurse interns, and service providers) and prepare and align staff on DMHI adoption (eg, consensus discussion) during the exploration stage [23].

Concerns about privacy violations have been reported in the literature as client-related barriers to implementing DMHIs [22,48]. Reducing the likelihood of privacy violations is key to trust building and inclusive care; clients want to ensure that they have control over their devices and data (eg, as opposed to having the app monitor them like a “the big brother” as previously described in the *Results* section) and that their data are protected and used for their designed clinical purpose. Increasing data transparency, allowing users to delete data from the app and server, and limiting data exchange with DMHI vendors may help build trust. DMHI products must be HIPAA compliant when applicable. Our data indicated that providers would be hesitant to introduce a DMHI to their clients if it does not have adequate privacy protection mechanisms in place.

Infrastructural problems, such as organizational characteristics in the inner context and leadership in the outer context, must be addressed to support DMHI implementation. Proper institutional resources, such as personnel, funding, and administrative support, are documented factors that influence the implementation success of DMHIs [24]. In our analyses, multiple providers mentioned that they did not have a work phone, waited a long time to get a test account, and did not have proper DMHI-specific training, which restricted providers from trying DMHIs themselves and consequently discouraged them from introducing DMHIs to their clients. Lattie et al [22] shared that even when providers have ample access to appropriate devices (eg, office space and work phones), strict rules and regulations can still restrict them from integrating DMHIs into their clinical practice, suggesting that additional attention is needed to ensure clear regulation around work-based technology with reasonable flexibility. In addition to supporting providers with necessary tools and appropriate regulations, providing proper provider training, including broader training about digital mental health technology and specific training related to the selected DMHI and its features, is also important.

Ongoing efforts are needed to address infrastructural problems at the system level, as the current staffing structure and payment models in the US health care sector do not provide the most nurturing environment for DMHI implementation [12]. In our analysis, we identified main provider concerns regarding (1) competing work demands and high workloads that contributed to providers’ resistance to DMHIs and introducing them to clients and (2) inability to bill for using DMHIs to interact with clients or manage information. Although DMHIs are often intended to increase the efficiency of clinical care, it should be acknowledged that sometimes DMHIs could add new work to busy providers, especially when the benefits of specific DMHI features and related regulations are unclear to the providers. Additional funding and resources are needed to support proper training and communication, such as reimbursing clinical staff’s time spent on learning about, introducing, and using DMHIs and sharing knowledge with colleagues about different DMHIs.

### Limitations and Future Directions

This paper presents the qualitative analyses of 69 interviews from 6 California counties participating in a single state-funded project, limiting the generalizability of the findings. Although we collected interview data from multiple California counties that vary in geographic locations and client sociodemographic characteristics, only 1 participating county (Modoc County) is considered rural based on the most recent US Census Bureau data [39]. Therefore, we were limited in our ability to analyze rural-urban differences in the factors influencing implementation. The generalizability of the findings to other states in the United States or other countries is limited. Furthermore, Help@Hand is an innovation project supported by funding specifically appropriated by California’s Mental Health Service Act, which is a unique case that may not be replicable in other contexts; thus, implementing a similar project in other states would require funding considerations. Moreover, personal identifying information, such as demographic information and training background, was not collected from the interviewees. Many of the early and postimplementation interviews were not completed by the same interviewees, as illustrated by the different types of roles that the interviewees held (shown in Table 1). Thus, we cannot comment on the diversity or training background of the participants or compare the interviewees’ responses in the early and postimplementation interviews. However, the sample size of the study (69 interviews) is relatively large for qualitative work, and in many settings, we were able to interview all or most of the clinical staff. Furthermore, all interviews were conducted with service providers, clinical supervisors, peers, community health workers, nurse interns, and leadership. Although we reported on some client-level barriers and facilitators (eg, digital literacy and access to technology), such perspectives were not directly solicited from clients themselves. Thus, we may have missed important client-level facilitators, barriers, and recommendations, as clients may not share all their concerns openly with clinical staff and not all factors (in particular, challenges before accessing a provider) were observable by providers. Gathering client perspectives and understanding their lived experiences are critical to the success of DMHI implementation. Our interviews were mostly conducted from 2018 to 2020, and only a few interviews were conducted during the COVID-19 pandemic. Owing to massive shifts in service delivery models and increased use of DMHIs during the pandemic [28,29], clinical staff’s perspectives and attitudes toward DMHIs may have changed accordingly. The results presented should be interpreted in the prepandemic and early pandemic contexts. A future study could be conducted during the postpandemic period to further understand the unique facilitators of and barriers to DMHI implementation during public health crises.

The limitations discussed above point to directions for future research. First, the analyses reported in this paper could be greatly enhanced using a mixed methods approach. For example, survey data and quantitative analyses could provide additional information on the impact of providers’ sociodemographic and training backgrounds on the implementation of DMHIs, particularly on the perceived clinical utility and fit of DMHIs. Repeated quantitative surveys may offer opportunities to understand the longitudinal changes in clinician attitudes and sentiments from before to during and after DMHI implementation. However, the clinician burden related to research participation (eg, the amount of time needed to fill out research questionnaires) may impede successful implementation. In addition, future work is needed to generate insights into client perspectives that are the most relevant to the day-to-day lived experiences of clients and their caregivers. For example, involving clients who vary in their age and racial and ethnic, language, and disability backgrounds in future studies may help us better understand how to tailor the design and content of DMHI features (innovation readiness) to improve their usefulness and usability and meet the needs, preferences, and constraints of diverse clients. Client perspectives are also important for understanding infrastructural problems and system factors, especially with regard to client hesitancy to use professional care. For example, clients may avoid seeking county behavioral health services owing to self-stigma (individual level) and societal stigma (system level). Further outreach and analyses of client perspectives would shed light on unique barriers and facilitators related to these historically marginalized client groups.

### Conclusions

This study involving 69 interviews provides insights into the barriers, facilitators, and recommendations related to DMHI implementation, which are salient to the clinical staff and leadership from counties participating in a state-funded project, Help@Hand, in California, United States. Increasing access to technology, improving digital literacy, improving the fit of DMHIs with current clinical practices and client populations, and addressing system-level barriers were found to be critical factors for implementation success. As mentioned earlier, conceptualizing DMHIs as services, rather than products, helps highlight the need to address the identified barriers while DMHIs are integrated into various care settings [19]. Conceptualizing DMHIs as services encourages evaluation above and beyond important innovation characteristics (eg, efficacy, safety, and clinical usefulness) and highlights the importance of evaluating the ecosystem around DMHIs, such as individual, organizational, and system-level factors; the fit between the innovation and implementation settings; and the processes necessary to integrate DMHIs into workflows and routine care settings. Understanding the ecosystem and infrastructural factors would allow us to support decision-making around selecting and implementing usable and helpful tools that can meet the needs of clients and providers across inner and outer contexts. We conclude that despite the promise of DMHIs, their potential can be severely limited if the barriers are not properly addressed and the facilitating conditions are not in place or not fully leveraged. As our data also captured recommendations for improvement strategies offered by the various invested parties, we hope that these findings, in particular, those related to engaging marginalized clients, improving digital literacy, improving innovation fit, and facilitating infrastructural changes, will be useful to other systems and settings that are attempting to implement DMHIs.

## Appendix

Multimedia Appendix 1 Interview guide.

Multimedia Appendix 2 Frequency and percentage of the codes by Exploration, Preparation, Implementation, Sustainment domains.

## Abbreviations

DHMI: digital mental health intervention
EPIS: Exploration, Preparation, Implementation, Sustainment
HIPAA: Health Insurance Portability and Accountability Act

## Data Availability

The data collected and analyzed for this study are not publicly available because this was not a requirement of the project’s funder, and the verbal consent process did not elicit participants’ consent for their data to be publicly shared.
