# Supplementary material for: Facilitators of and Barriers to Integrating Digital Mental Health Into County Mental Health Services: Qualitative Interview Analyses
Source: JMIR Form Res. 2023 May 16;7:e45718. doi: 10.2196/45718 (PMC10230355; doi:10.2196/45718)
Supplement: Multimedia Appendix 1 [file formative_v7i1e45718_app1.docx]

Appendix 1. Interview Guide.

**Provider Questions**

*“Thank you for taking the time to meet with us today. We are part of the UC Irvine Evaluation Team helping understand the implementation of [BLANK] County Innovation Tech Suite [Help@Hand]. We’re interested in learning more about the process of implementing [PRODUCT/PRODUCTS] over the past 6 months. We would like to gather your perspectives through this interview that will take about 30 minutes. We would like to audio-record our interview with you so that our evaluation team can review and synthesize what you shared. Please know that we will not share your individual responses with your supervisor or [COUNTY]. There are no right or wrong answers. You are the expert. We are here to understand your perspectives and experiences, so we will not be sharing our opinions. Do you have any questions before we turn on the recorder and begin?”*

[TURN ON AUDIORECORDER]

“*Great! Let’s start with me asking you some general questions about your work as a provider and any experience with mental health technology.”*

A. “How long have you been working as a provider in [COUNTY]?”

B. “What are the types of clinical services do you provide?”

C. “How familiar are you with [PRODUCT]?

*“We would like to start our interview with general questions about your experiences with using technology in your work.”*

1. Prior to [PRODUCT] being used here, had you or your clients used mobile apps in treatment?
   1. [FOLLOWUP IF NO:] Why hadn’t you used mobile apps in your practice?
   2. [FOLLOW UP IF YES:] Which apps and what did they help you do? How did they impact the treatment you provided?
   3. [FOLLOW UP IF YES:] What challenges have you encountered using apps besides [PRODUCT] in treatment?

*“Now we would like to ask more specific questions about your experiences with [PRODUCT], remember we’re interested in learning more about the impact [PRODUCT] has had on this clinic and your practices over the past 6 months and [PRODUCT]’s continued use in this clinic.”*

1. Before [PRODUCT] started to be used in your clinic, what were your expectations of it?
   1. PROBE: Which of those expectations were met?
   2. PROBE: How have those expectations changed?
2. With regards to [PRODUCT], what has been going well so far?
3. With regards to [PRODUCT], what has *not* been going well so far?
   1. What barriers made it hard for you to use [PRODUCT]? (e.g., concerns about privacy, validity, client safety)
4. What kinds of changes or “work-arounds” have you had to make to [PRODUCT] so it would work effectively in your setting?
   1. What changes or “work-arounds” did you make in terms of how [PRODUCT] was presented to clients?
   2. What additional materials or information did you provide to help clients use [PRODUCT]?
   3. What changes or “work-arounds” did you make in terms of how [PRODUCT] was used in sessions?
   4. What changes or “work-arounds” did you make to your work routine/workflow?
5. What features of [PRODUCT] do you like?
   - 1. How does this compare to the existing way you monitor progress or changes in your practice?
     2. What about the [FEATURE] did you find helpful?
     3. What about the [FEATURE] did you *not* find helpful?
6. Is there another intervention that people would rather implement?
   1. Can you describe that intervention?
   2. Why would people prefer the alternative?
7. How have your clients served responded to [PRODUCT]?

[NOTE DON’T NEED TO ASK ALL THESE, BUT PROBE FOR POSITIVES AND NEGATIVES]

- 1. Have you had clients that have refused to use [PRODUCT]? Why or why not?
  2. Have you had clients that have experienced challenges using [PRODUCT]? What have been some of those challenges?
  3. Have you had clients who have expressed benefit to [PRODUCT]?
  4. Do your patients need additional support to use [PRODUCT]? If so, what additional support might they need?
  5. Has [PRODUCT] changed your relationship with your patients? If so, how?

1. Did you discontinue using [PRODUCT] with any of your clients?
   1. If so, what factors contributed to your discontinuing use of [PRODUCT]?

9b. Did any of your clients discontinue using [PRODUCT]?

- 1. If so, what factors contributed to their discontinued use of [PRODUCT]?

1. What has been the general level of receptivity here to implementing [PRODUCT]?
   1. Why?
2. Who have been the key champions during this implementation of [PRODUCT]?
   1. What have they done to support the implementation of [PRODUCT] here?
3. How confident have you been during your use of [PRODUCT] thus far?
   1. What gives you that level of confident (or lack of confidence)?
   2. How confident are you to continue your use of [PRODUCT]?
      1. If high – what gives you that confidence?
      2. If low – what would help you increase that confidence?
4. Do you think [PRODUCT] has been effective in your setting?
   1. Why or why not?

*“Thank you for taking the time to speak with me today! Your perspectives are very valuable to our understanding of the Tech Suite evaluation.”*

**Leadership Questions**

*“Thank you for taking the time to meet with us today. We are part of the UC Irvine Evaluation Team helping understand the implementation of [COUNTY] Innovation Tech Suite [Help@Hand]. We’re interested in learning more about the process of implementing [PRODUCT] over the past 6 months. We would like to gather your perspectives through this interview that will take about 30 minutes. We would like to audio-record our interview with you so that our evaluation team can review and synthesize what you shared. Please know that we will not share your individual responses with your supervisor or [COUNTY]. There are no right or wrong answers. You are the expert. We are here to understand your perspectives and experiences, so we will not be sharing our opinions. Do you have any questions before we turn on the recorder and begin?”*

[TURN ON AUDIORECORDER]

*“Great! Let’s start with me asking you some general questions about your work as a provider and any experience with mental health technology.”*

A. “How long have you been working in [COUNTY]?”

B. “What is your role and what type of activities do you do in this role?”

C. “How familiar are you with [PRODUCT]?

1. Prior to [PRODUCT] being used here, had you or your clients used mobile applications, or apps, in treatment?
   1. [FOLLOWUP IF NO:] Why hadn’t you used mobile apps in your practice?
   2. [FOLLOW UP IF YES:] Which apps and what did they help you do? How did they impact the treatment you provided?
   3. [FOLLOW UP IF YES:] What challenges had you encountered using mobile apps besides [PRODUCT]?

*“Now we would like to ask more specific questions about your perceptions of using [PRODUCT] in your county (or clinic/setting).”*

1. Before [PRODUCT] started to be used in your clinic, what were your expectations of it?
   1. PROBE: Which of those expectations were met?
   2. PROBE: How have those expectations changed?
2. With regards to [PRODUCT], what has been going well so far?
3. With regards to [PRODUCT], what has *not* been going well so far?
   1. What barriers made it hard for you to use [PRODUCT]?
4. What kinds of changes or “work-arounds” were made so [PRODUCT] would work effectively in your setting?
   1. What changes or “work-arounds” did you make in terms of how [PRODUCT] was presented to clinicians?
   2. What changes or “work-arounds” were made in terms of training and supervision?
   3. What changes or “work-arounds” were made in terms of clinician time or responsibilities?
5. What kinds of infrastructure changes have you made to accommodate [PRODUCT]?
   1. Changes in scope of practice? Changes in formal policies? Changes in information systems or electronic records systems? Other?
   2. What kind of approvals were needed? Who needed to be involved?
6. How have these changes gone?
   1. Which of these changes have been helpful?
   2. Which of these changes would you continue?
   3. What additional changes are necessary? (Are you planning to make those changes?)
7. What has been the general level of receptivity here to implementing [PRODUCT]?
   1. Why?
8. What kinds of high-priority initiatives or activities have been happening in your setting?
   1. What is the priority of getting [PRODUCT] implemented relative to other initiatives that are happening now?
9. How have the clients served here responded to [PRODUCT]?
10. Do you think [PRODUCT] has been effective in your setting?
    1. Why or why not?
11. How likely are you to continue to use [PRODUCT] here?
    1. If high – what contributes to that likelihood?
    2. If low – what would you need to become more likely to continue use?

*“Thank you for taking the time to speak with me today! Your perspectives are very valuable to our understanding of the [COUNTY] Innovation Tech Suite [Help@Hand] evaluation.”*
