# Supplementary material for: Facilitators of and Barriers to Integrating Digital Mental Health Into County Mental Health Services: Qualitative Interview Analyses
Source: JMIR Form Res. 2023 May 16;7:e45718. doi: 10.2196/45718 (PMC10230355; doi:10.2196/45718)
Supplement: Multimedia Appendix 2 [file formative_v7i1e45718_app2.docx]

Appendix 2. Frequency and percentage of the codes by EPIS domains

|  | Facilitators | | | Barrier | | | Recommendation | | |
| --- | --- | --- | --- | --- | --- | --- | --- | --- | --- |
| **EPIS codes** | Frequency | % | n of counties in which codes were mentioned | Frequency | % | n of counties in which codes were mentioned | Frequency | % | n of counties in which codes were mentioned |
| Inner Context |  |  |  |  |  |  |  |  |  |
| Individual characteristics | 251 | 25.35% | 6 | 142 | 14.61% | 6 | 17 | 6.03% | 5 |
| Organization Characteristics | 165 | 16.67% | 6 | 171 | 17.59% | 6 | 41 | 14.54% | 6 |
| Leadership | 23 | 2.32% | 3 | 8 | 0.82% | 4 | 2 | 0.71% | 2 |
| Quality and Fidelity Monitoring/Support | 11 | 1.11% | 5 | 7 | 0.72% | 4 | 15 | 5.32% | 5 |
| Organization Staffing process | 1 | 0.10% | 1 | 4 | 0.41% | 3 | 1 | 0.35% | 1 |
| Innovation Factor |  |  |  |  |  |  |  |  |  |
| Innovation Characteristics | 193 | 19.49% | 6 | 186 | 19.14% | 6 | 34 | 12.06% | 6 |
| Innovation Fit | 93 | 9.39% | 6 | 80 | 8.23% | 6 | 29 | 10.28% | 5 |
| Innovation Developers | 10 | 1.01% | 2 | 10 | 1.03% | 5 | 88 | 31.21% | 6 |
| Outer Context |  |  |  |  |  |  |  |  |  |
| Client Characteristics | 143 | 14.44% | 6 | 232 | 23.87% | 6 | 15 | 5.32% | 4 |
| Client Advocacy | 23 | 2.32% | 6 | 18 | 1.85% | 4 | 8 | 2.84% | 3 |
| Leadership | 6 | 0.61% | 3 | 21 | 2.16% | 4 | 9 | 3.19% | 2 |
| Inter Organizational Environment and Networks | 12 | 1.21% | 3 | 11 | 1.13% | 2 | 2 | 0.71% | 2 |
| Funding/contracting | 0 | 0.00% | 0 | 6 | 0.62% | 2 | 0 | 0.00% | 0 |
| Service environment | 0 | 0.00% | 0 | 24 | 2.47% | 5 | 5 | 1.77% | 1 |
| Bridging Factors |  |  |  |  |  |  |  |  |  |
| Purveyors/Intermediaries | 33 | 3.33% | 5 | 17 | 1.75% | 3 | 10 | 3.55% | 4 |
| Community Academic Partnerships | 3 | 0.30% | 3 | 0 | 0.00% | 0 | 2 | 0.71% | 1 |
| Other | 23 | 2.32% | 5 | 35 | 3.60% | 5 | 4 | 1.42% | 3 |
| Total | 1,019 | 100% |  | 985 | 100% | - | 289 | 1 | - |
